# Supplementary material for: Mechanistic characterization of a Drosophila model of paraneoplastic nephrotic syndrome
Source: Nat Commun. 2024 Feb 9;15:1241. doi: 10.1038/s41467-024-45493-8 (PMC10858251; doi:10.1038/s41467-024-45493-8)
Supplement: Supplementary file 1 — Supplementary Information [file 41467_2024_45493_MOESM1_ESM.docx]

**Mechanistic characterization of a *Drosophila* model of paraneoplastic nephrotic syndrome**

Jun Xu^1, 2 #,^ *, Ying Liu^2, #,^ *, Fangying Yang^1^, Yurou Cao^1^, Weihang Chen^2^, Joshua Shing Shun Li^2^, Shuai Zhang^1^, Aram Comjean^2^, Yanhui Hu^2^, Norbert Perrimon^2, 3,^ *

^1^ CAS Key Laboratory of Insect Developmental and Evolutionary Biology, CAS Center for Excellence in Molecular Plant Sciences, Shanghai Institute of Plant Physiology and Ecology, Chinese Academy of Sciences, China.

^2^ Department of Genetics, Blavatnik Institute, Harvard Medical School, Boston, Massachusetts, USA.

^3^ Howard Hughes Medical Institute, Boston, Massachusetts, USA.

^#^ These authors contributed equally

*Correspondence: junxu@cemps.ac.cn; ying_liu@hms.harvard.edu; perrimon@genetics.med.harvard.edu.

**Supplementary Information**

Supplementary Figures 1-9

Supplementary Table 1


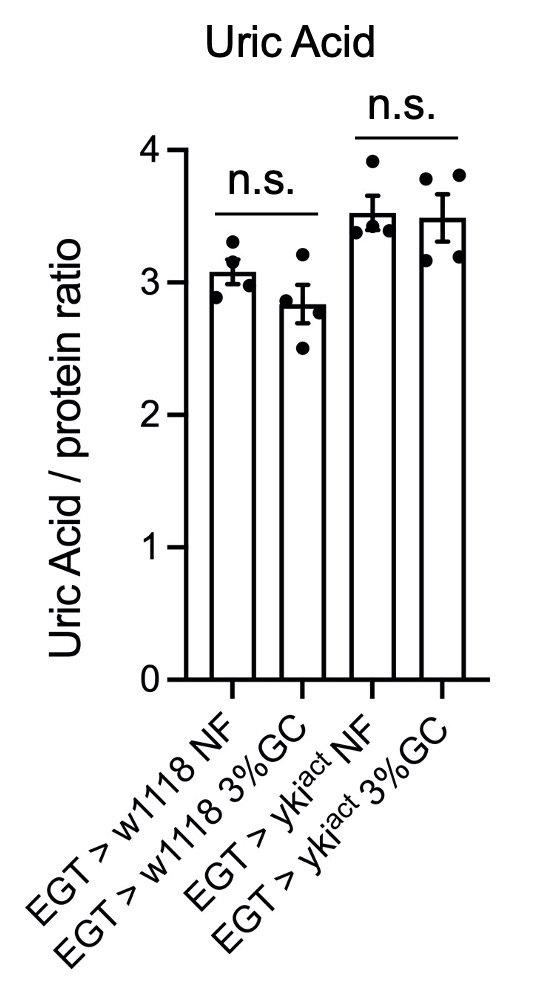


**Supplementary Figure 1.** (A) Whole-body levels of uric acid in *EGT > w1118* and *EGT > yki^act^* flies at 8 days. Flies were fed either normal food or food with 3% *Garcinia cambogia* (GC). n = 4 biologically independent experiments. Data are presented as means ± SEM. n.s. means no significant with student t-test.

**
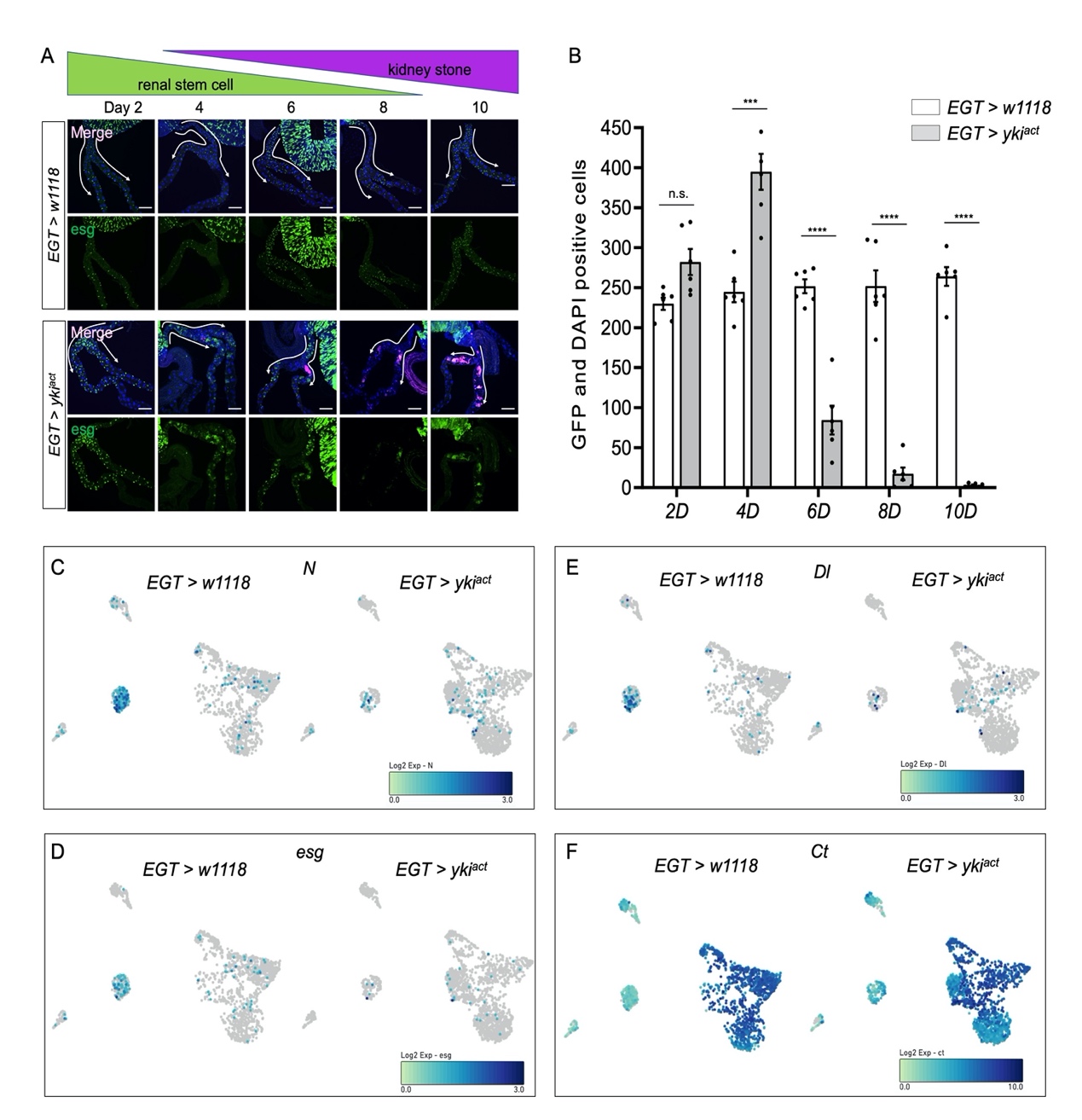
**

**Supplementary Figure 2. Aberrant renal stem cells renewal in flies with yki^act^ gut tumor.** (A) esg positive renal stem cell signal decreased during the growth of yki^act^ gut tumor. Green indicates esg positive cells, blue is DAPI staining. White arrows indicate the renal stem cell zone. (B) Changes in renal stem cells number during the growth of yki^act^ gut tumor. n = 6 biologically independent experiments. Data are presented as means ± SEM. ***p < 0.001, ****p < 0.0001. n.s. means no significant with student t-test. N=6 for each column. (C-F) Notch (N) signaling was decreased in the Malpighian tubule in yki^act^ gut tumor flies. C-E show the decrease in the UMAP of *N* expression and its two downstream genes, *esg* and *Dl*. F show the decrease in the UMAP of *ct* expression in principal cells.

**
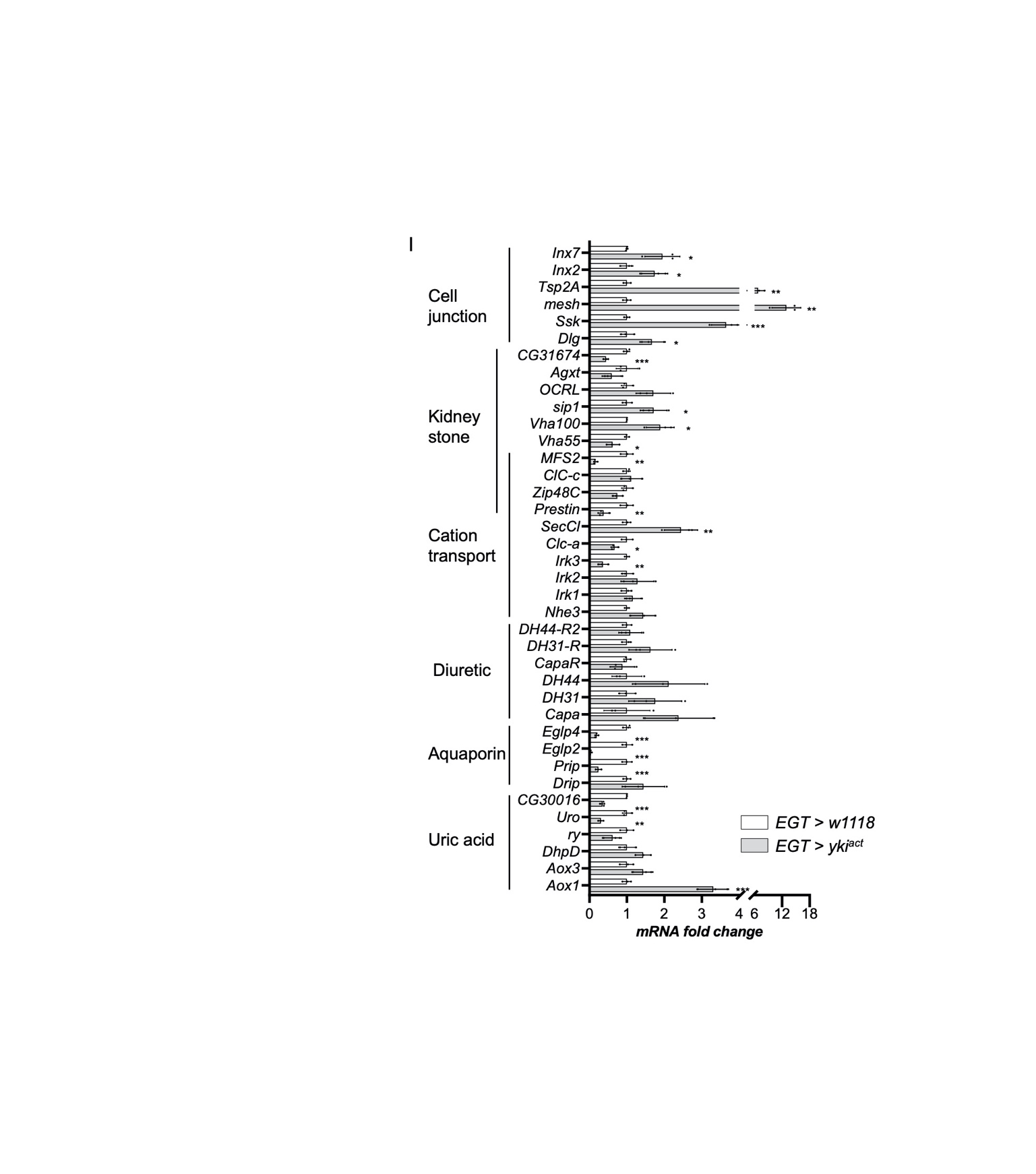
**

**Supplementary Figure 3. Changes in the expression of genes involved in MT function.** (A) Function of principal and stellate cells in MT. Adapted from Xu et al., 2022. (B-G) Violin plots showing the expression of genes involved in kidney function (cell junction, kidney stone risk, cation transport, diuretic, aquaporin, uric acid) in the different cell clusters between *EGT > w1118* and *EGT > yki^act^*. (H) Model of renal dysfunction based on the expression of genes involved in uric acid synthesis, kidney stone formation and water transport in *yki^act^* flies. (I) qPCR analysis indicating the changes in gene expression levels in the MT of *EGT > w1118* and *EGT > yki^act^*. Transgene expression was induced for 8 days. n = 3 biologically independent experiments with 3 technical replicates. Data are presented as means ± SD. *p < 0.05, **p < 0.01, ***p < 0.001 with student t-test.


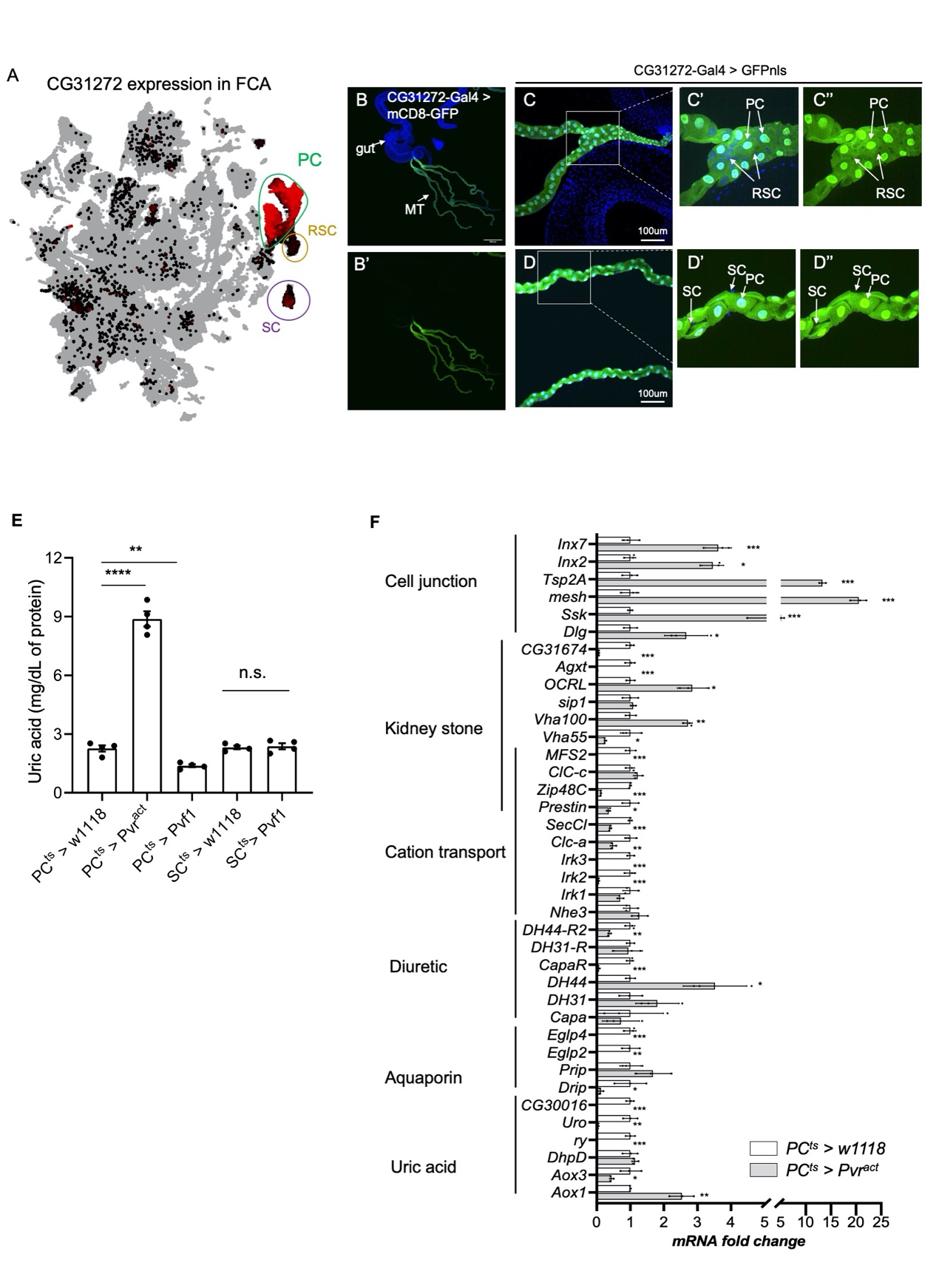


**Supplementary Figure 4.** (A) FCA cell clusters with red indicates CG31272 expression. CG31272 is highly expressed in principal cells (PCs), and with a much lower expression level in renal stem cells (RSC) and stellate cells (SC) (data obtained from the FCA dataset; Li et al., 2022). (B-D) Characterization of a new Gal4 driver for MT principal cells (PCs). (E) Whole-body UA level in the *PC^ts^ > Pvr^act^, Pvf1, and SC^ts^ > Pvr^act^, Pvf1*, and control flies, n = 4 biologically independent experiments. (F) qPCR analysis indicating the changes in gene expression levels in the MT of *PC^ts^ > w1118* and *PC^ts^ > Pvr^act^*. Transgene expression was induced for 8 days. n = 3 biologically independent experiments with 3 technical replicates. Data are presented as means ± SD. *p < 0.05, **p < 0.01, ***p < 0.001 with student t-test.

**Supplementary Figure 5.** Western blots indicating the protein level of ERK and phosphorylated ERK in the MTs of **(A)** *EGT > w1118* and *EGT > yki^act^* and **(B)** *PC^ts^ > w1118* and *PC^ts^ > Pvr^act^* flies. Transgenes were induced for 8 days.


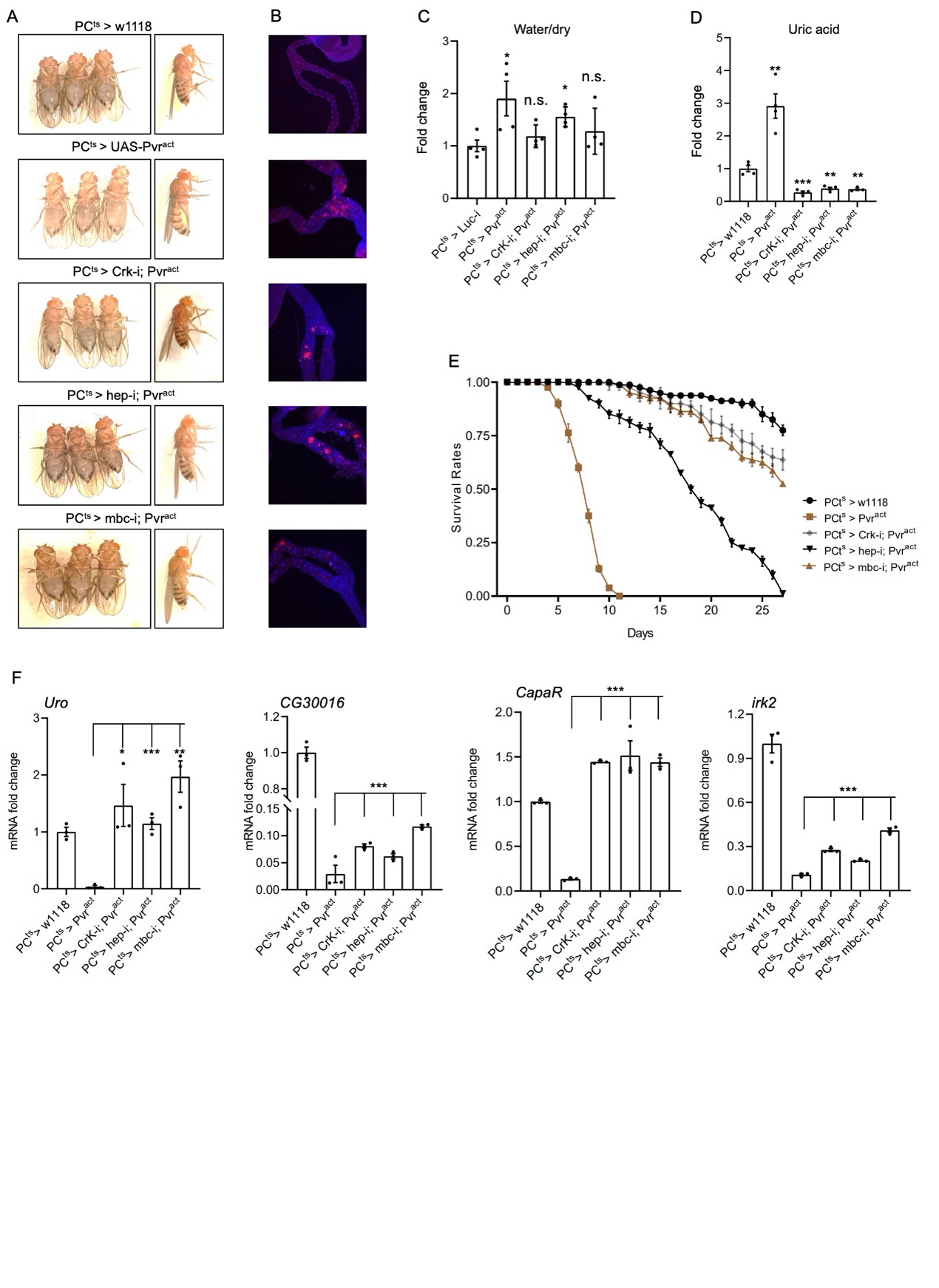


**Supplementary Figure 6. Inhibition of PDGF/VEGF signaling components in principal cells can rescue MT dysfunction associated with Pvract.** (A) Bloating phenotypes associated with expression of *Pvr^act^, Crk-i; Pvr^act^, hep-i; Pvr^act^* and *mbc-i; Pvr^act^* , driven by PC^ts^ in principal cells. (B) Kidney stone phenotypes: blue is for DAPI staining to detect nuclei and purple indicates the kidney stones. (C) Ratio of fly water/dry mass, n = 4 biologically independent experiments. (D) Whole-body UA level, n = 4 biologically independent experiments. (E) Lifespan, n = 80 for each genotype. (F) qPCR results showing changes in gene expression levels in the MT. n = 3 biologically independent experiments with 3 technical replicates. Data are presented as means ± SEM. *p < 0.05, **p < 0.01, ***p < 0.001, ****p < 0.0001. n.s. means no significant with student t-test.


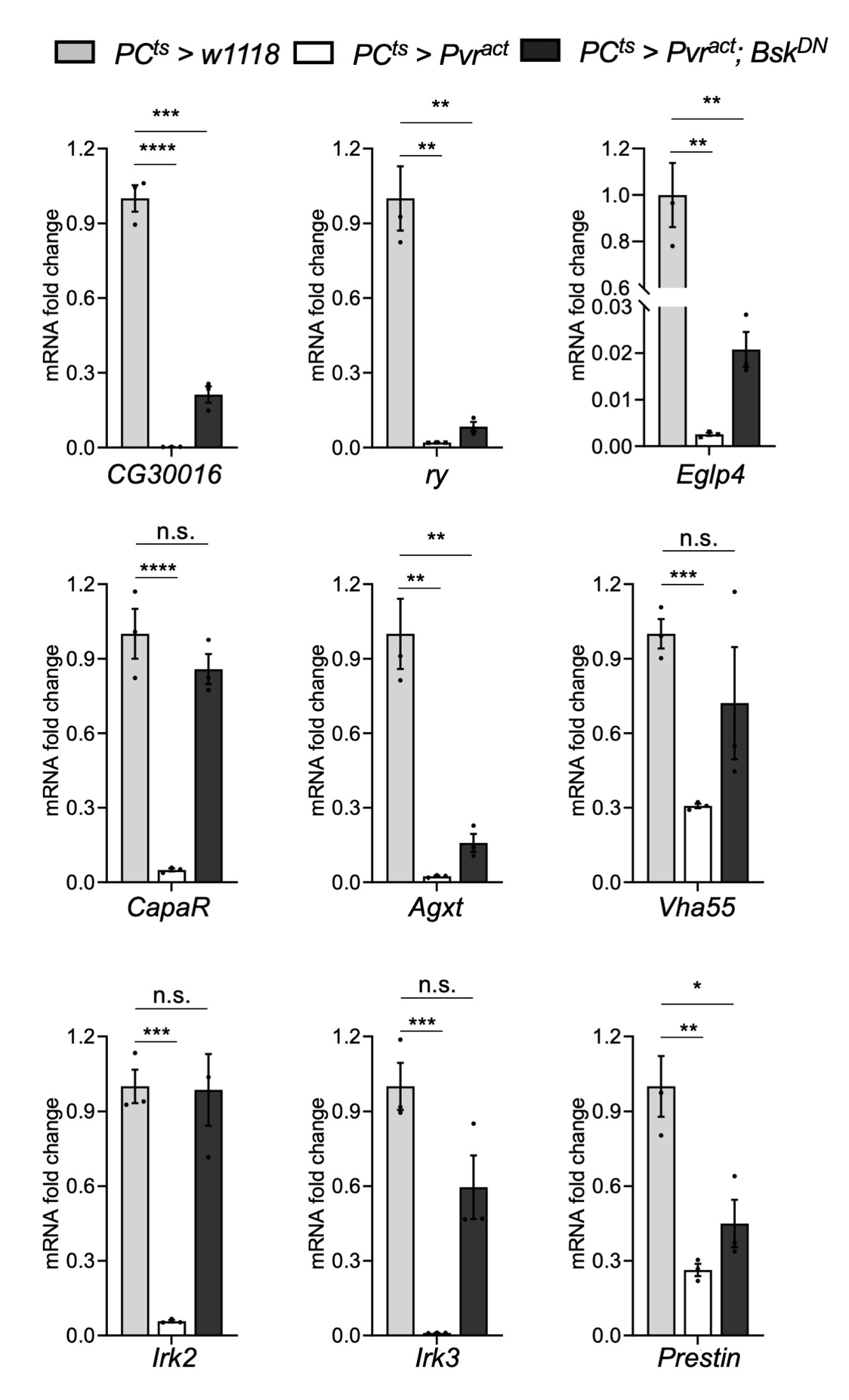


**Supplementary Figure 7. Expression of genes involved in kidney function.** qPCR results showing changes of gene expression level in the MTs of *PC^ts^ > Pvr^act^*, *PC^ts^ > Pvr^act^; Bsk^DN^* and control flies. n = 3 biologically independent experiments with 2 or 3 technical replicates.


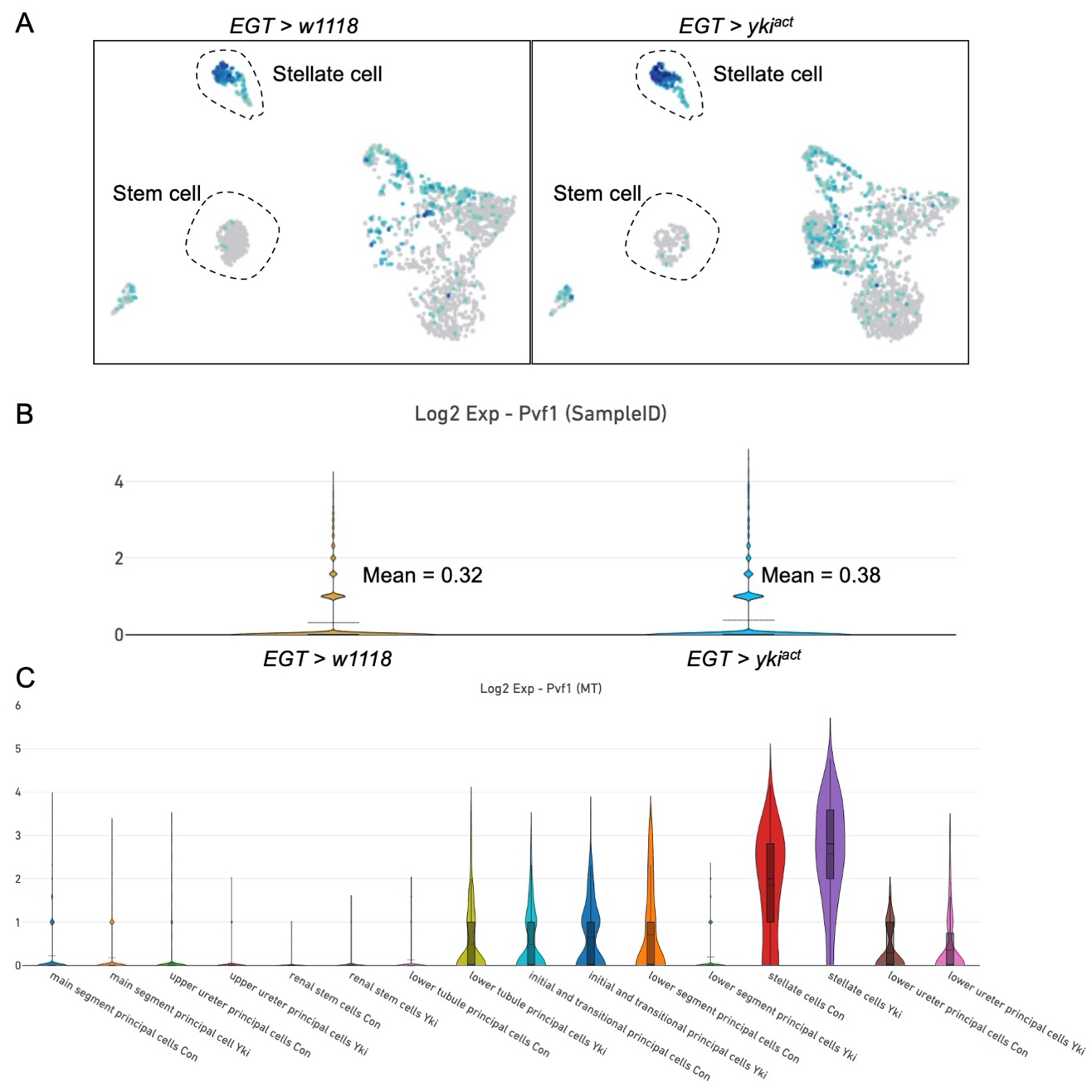


**Supplementary Figure 8. *Pvf1* expression in the MT cell cluster.** (A) Gene expression of *Pvf1* in the UMAP. Dotted box showed the stellate cell and stem cell. (B) and (C) Expression levels of *Pvf1* in *EGT > w1118* and *EGT > yki^act^* MTs visualized by violin plots in (B) all MT cells and (C) each cluster, respectively.

**
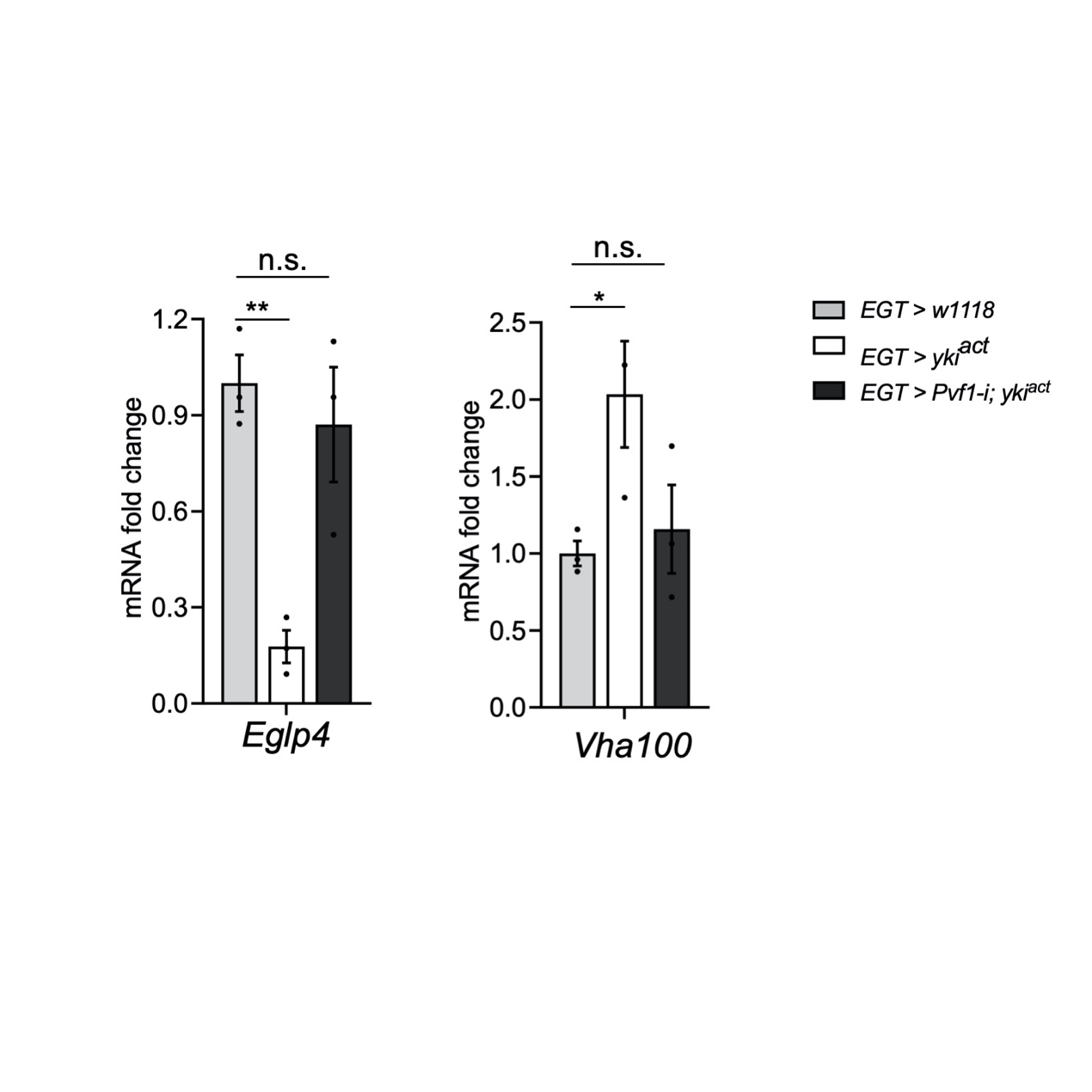
**

**Supplementary Figure 9. Expression of genes involved in kidney function.** qPCR results showing changes in *Eglp4* and *Vha10*0 expression levels in the MTs of Yki flies with and without ISC depletion of Pvf1 and control flies. n = 3 biologically independent experiments with 3 technical replicates.

| **FlyBase ID** | **Symbol** | **Log2FC Pvr-RNAi vs Ctrl** | **Log2FC Pvr-act vs Ctrl** | **Log2FC Yki vs Ctrl** |
| --- | --- | --- | --- | --- |
| FBgn0038652 | CG7720 | 1.9746 | -1.7629 | 1.6363 |
| FBgn0027556 | CG4928 | 0.5734 | -3.0777 | -0.0284 |
| FBgn0012036 | Aldh | 0.6186 | -0.7477 | -0.0602 |
| FBgn0037873 | SdhC | 0.5953 | -0.8853 | -0.0866 |
| FBgn0003866 | tsh | 0.5409 | -2.0270 | -0.2657 |
| FBgn0033665 | Zip48C | 0.8474 | -1.2666 | -0.2837 |
| FBgn0032833 | COX4 | 0.5175 | -1.4263 | -0.2917 |
| FBgn0265042 | Irk1 | 1.4766 | -2.3004 | -0.3302 |
| FBgn0029131 | Debcl | 0.5347 | -1.2512 | -0.4605 |
| FBgn0035695 | CG10226 | 0.5687 | -0.5766 | -0.5608 |
| FBgn0039872 | salt | 0.7417 | -1.5874 | -0.5694 |
| FBgn0031523 | CG15408 | 0.5121 | -1.4362 | -0.5803 |
| FBgn0262512 | Vha14-1 | 0.4493 | -1.4397 | -0.5941 |
| FBgn0027844 | CAH1 | 0.5603 | -2.5268 | -0.5969 |
| FBgn0031517 | CG15406 | 1.1491 | -2.1363 | -0.6044 |
| FBgn0039311 | CG10513 | 0.7089 | -2.7747 | -0.9527 |
| FBgn0052656 | Muc11A | 0.7755 | -3.1731 | -0.9913 |
| FBgn0038337 | CG6125 | 0.6055 | -1.4366 | -1.0310 |
| FBgn0039312 | CG10514 | 1.2411 | -2.1127 | -1.0723 |
| FBgn0051163 | SKIP | 0.7530 | -1.2103 | -1.3229 |
| FBgn0051373 | CG31373 | 0.7249 | -2.8435 | -1.3588 |
| FBgn0025454 | Cyp6g1 | 0.5251 | -2.7305 | -2.2934 |
| FBgn0267408 | AOX1 | 0.2625 | 0.6094 | 1.1087 |
| FBgn0003961 | Uro | -0.2996 | -3.3012 | -1.3586 |
| FBgn0034883 | Eglp2 | 0.2974 | -1.4201 | -1.2448 |
| FBgn0016684 | MFS2 | -0.3687 | -2.3499 | -1.4559 |
| FBgn0051004 | mesh | -0.1032 | 1.7417 | 1.3608 |
| FBgn0024361 | Tsp2A | -0.0851 | 1.6834 | 1.3441 |

**Supplementary Table 1. snRNAseq of flies with renal PDGF/VEGF signaling activation or inhibition.** Expression alternations of selected genes, full list sees Supplementary Data 2.
