# Supplementary material for: Mechanistic characterization of a Drosophila model of paraneoplastic nephrotic syndrome
Source: Nat Commun. 2024 Feb 9;15:1241. doi: 10.1038/s41467-024-45493-8 (PMC10858251; doi:10.1038/s41467-024-45493-8)
Supplement: Supplementary file 3 — Description of Additional Supplementary Files [file 41467_2024_45493_MOESM3_ESM.pdf]

Description of Additional Supplementary Files

Supplementary Data 1. Upregulated genes upon Pvr depletion in main segment principal cells. Top 50 upregulated genes Pvr_RNAi vs Ctrl in main segment principal cells were listed.

Supplementary Data 2. Differential expressed genes (DEGs) upon Pvr depletion and activation in all MT cells. DEGs in Pvr_RNAi and Pvr_act samples compared with ctrl were listed.

Supplementary Data 3. Primers used for qRT-PCR
